# Mesoporous Silica with an Alveolar Construction Obtained by Eco-Friendly Treatment of Rice Husks

**DOI:** 10.3390/molecules29153540

**Published:** 2024-07-27

**Authors:** Margarita Popova, Violeta Mitova, Momtchil Dimitrov, Consolato Rosmini, Ivelina Tsacheva, Pavletta Shestakova, Daniela Karashanova, Irina Karadjova, Neli Koseva

**Affiliations:** 1 Institute of Organic Chemistry with Centre of Phytochemistry, Bulgarian Academy of Sciences, 1113 Sofia, Bulgaria; momtchil.dimitrov@orgchm.bas.bg (M.D.); consolato.rosmini@orgchm.bas.bg (C.R.); pavletta.shestakova@orgchm.bas.bg (P.S.); 2 Institute of Polymers, Bulgarian Academy of Sciences, 1113 Sofia, Bulgaria; mitova@polymer.bas.bg (V.M.); itsacheva@polymer.bas.bg (I.T.); 3 Institute of Optical Materials and Technologies, Bulgarian Academy of Sciences, 1113 Sofia, Bulgaria; dkarashanova@yahoo.com; 4 Faculty of Chemistry and Pharmacy, Sofia University “St. Kliment Ohridski”, 1, James Bourchier Blvd.1, 1164 Sofia, Bulgaria; karadjova@chem.uni-sofia.bg; 5 Bulgarian Academy of Sciences, 1040 Sofia, Bulgaria; koseva@polymer.bas.bg

**Keywords:** rice husks, waste valorization, mesoporous silica

## Abstract

The high silicon content in rice plant waste, specifically rice husks, makes this waste by-product attractive for the extraction and valorization of silicon oxide, which is widely used as an inert support in catalysis, drug delivery and molecular sieving. The procedures currently used for the treatment of plant biomass make extensive use of mineral acids (HCl, H_2_SO_4_, HNO_3_), which, besides them being potential environmental pollutants, reduce the yield and worsen the chemical-physical properties of the product. In this study, an evaluation of the easy treatment of rice husks by benchmarking different, more eco-friendly carboxylic acids in order to obtain a mesoporous SiO_2_ with an alveolar structure and a relatively high surface area and pore volume (300–420 m^2^/g, 0.37–0.46 cm^3^/g) is presented. The obtained mesoporous silicas are characterized by worm-like pores with a narrow size distribution and a maximum in the range of 3.4–3.5 nm. The mesoporous structure of the obtained materials was also confirmed by TEM. The complete removal of the organic part of the rice husks in the final materials was evidenced by thermogravimetric analysis. The high purity of the obtained mesoporous silica was detected using ICP analysis (98.8 wt. %). The structure peculiarities of the obtained mesoporous silicas were also characterized by solid-state NMR and ATR-FTIR spectroscopies. The morphology of the mesoporous silica was investigated by SEM.

## 1. Introduction

The first procedure for the preparation of mesoporous silicas was described in 1992 by researchers of the Mobile company [1]. The structure of these materials is characterized by a well-defined system of hexagonally arranged channels. Mesoporous silicates have attracted the attention of the scientific community due to their unique properties, such as ordered pore structure, high specific surface area (700–1500 m^2^/g), large pore volume (0.7–1.2 cm^3^/g) and pore size varying in wide intervals (2–30 nm). The possibility to control the morphology and size of the particles (50 nm–1 μm) and their good mechanical, chemical and thermal stability are additional advantages of silica materials [2,3,4]. They are widely used as adsorbents, catalyst carriers, etc. [5]. Intensive research to develop new synthetic procedures allows the preparation of nanomaterials with desired morphology, particle size and textural characteristics, which predetermines their application in various catalytic processes for the production of important chemicals and as carriers of medicinal substances for treatment and diagnostics. The development of appropriate procedures for modifying mesoporous silicates with metal/metal oxides or organic functional groups is a prerequisite for their successful application.

The synthesis of mesoporous materials involves three main steps: synthesis, drying and template removal. The method and conditions used to remove the organic template affect the final total pore volume and size, but not their connectivity and arrangement. By choosing a suitable template with a certain concentration and in a certain ratio with the silicon precursor, materials with a certain pore size and structural arrangement are obtained. The most commonly used sources of silicon are tetraethylorthosilicate (TEOS) or tetramethylorthosilicate (TMOS) and sodium silicate. Modified silica mesoporous materials are promising catalysts for many processes due to their suitable structural and compositional characteristics. A disadvantage of these materials is their high cost, which is due to the use of expensive reagents, incl. templates, high temperatures and long procedures which lead to higher energy consumption.

A significantly more economical approach is the use of waste materials, lower temperatures and the absence of expensive templates to obtain mesoporous silicas. Examples of raw materials for obtaining porous materials are most often agricultural wastes, such as rice husks, etc. Annually, 80 million tons of rice husks are produced in the world, the main ingredients of which are organic substances such as cellulose, hemicellulose and lignin, as well as inorganic components, which, after burning the organic ones, are isolated as ash [6]. Fly ash consists mainly of silicon dioxide (SiO_2_) and some metal impurities. The recovery of SiO_2_ is the subject of considerable research interest due to its use as a raw material in the industry. In these studies, rice husks were treated with sulphuric acid, hydrochloric acid and nitric acid to remove alkali and alkaline earth metal impurities. The use of mineral acids could be associated with a negative effect on the environment, the use of expensive equipment with corrosion resistance, large volumes of water for rinsing the scales and special treatment of the spent wastewater. The use of carboxylic acids has a sparing effect on the environment and is economically more profitable. It is a challenge to develop a method for the direct preparation of mesoporous silica with a narrow pore size distribution from rice husks without using concentrated mineral acids, which implies the development of new strategies for processing the silica sources.

A large number of patents, for example, patent application CN102190301 (A), claim that high-purity silica can be obtained from rice husks via a procedure that involves several steps. For example, it may include an incineration step followed by subsequent purification steps of the resulting ash to remove any impurities present (sand particles, alkali metal compounds and residual carbon). It is known from the non-patent literature that the combustion of untreated rice husks at 600 °C for 5 h produces a white powdery material with a mesoporous structure, with a specific surface area of 36.9 m^2^/g and a pore volume of 0.094 cm^3^/g [7]. An alternative approach is also proposed in patent application CN1063087 (A), which involves treating rice husks with a mixture of concentrated nitric acid and 30% concentrated hydrogen peroxide under hydrothermal conditions below 150 °C, followed by natural cooling to room temperature and washing to neutral deionized water condition.

Nanosilicate materials were obtained by pretreating rice husks with Averrhoa juice bilimbi (1 h at 80 °C) and the subsequent calcination in a muffle furnace (500 °C for 6 h). The specific surface of the obtained materials is 204 m^2^/g [8].

Silica nanoparticles with controllable surface areas and pore volumes were obtained from rice husks pretreated with an HCl solution (8 wt. %) and subsequent heat treatment by pre-pyrolysis combined with calcination. The obtained materials are characterized by a specific surface area of 204–352 m^2^/g and a total pore volume of 0.35–0.52 cm^3^/g [9].

Other authors reported the preparation of mesoporous materials (Table 1) with a specific surface area in the range of 46 to 264 m^2^/g, after treatment of rice husks with hydrochloric acid (1 M HCl, 80 ° C, 2 h) and calcination in a muffle furnace at 500 °C for 4 h [10].

Biogenic silica is obtained from rice husks using gluconic and citric acids (at 80 °C for 2 h, at acid concentrations 0; 0.05; 0.15; 0.25; 0.35 mol/L) and subsequent combustion (at 800 °C for 2 h). The resulting mesoporous materials are characterized by a specific surface area of 114.06 m^2^/g and a pore volume of 0.23 cm^3^/g [11].

Amorphous silica nanoparticles were synthesized by the pyrolysis of rice husks residue at 700 °C for 2 h [12]. The rice husk was pretreated using ionic liquid (1-butyl-3-methylimidazolium chloride) within the interval of 100–150 °C for 12–36 h and with 5.0 wt. % HCl for 1 h. The surface area of the silica nanoparticles based on the acid-treated rice husks is slightly higher than that of the nanoparticles treated by the ionic liquid, 283.3 m^2^/g and 241.1 m^2^/g, respectively.

Several reviews show the recent progress with the production and utilization of the silica obtained from rice husks, especially its potential application as a catalytic support [6,14,15,16,17].

**Disadvantages of the existing methods for synthesis of mesoporous silicas:**

− Relatively low specific surface area and formation of pores with a wide size distribution of the obtained silica material, if the template is not used;
− Employment of multi-step, energy-intensive procedures or use of strong mineral acids containing halogens or groups such as nitrates or sulphates, which make the scale-up of the process expensive and potentially polluting wastewaters.

**In this study** a procedure is proposed for the easy preparation of mesoporous silicates with a narrow pore size distribution and a relatively high surface area with respect to the actual state of the art for these materials, screening a wide variety of carboxylic acids with low economic and environmental impact. The role of a bio-template effect reflected in the surface properties of the obtained materials has also been discussed.

## 2. Results and Discussion

With the aim of evaluating the effects brought about by the treatment of rice husks with carboxylic acids compared to the typical procedure using mineral acids, the vegetal matrices were also treated with nitric acid. After the acid treatment, the dry vegetable residue treated with HNO_3_ showed weight loss compared to the initial material of approximately 64.8%, while, when treated with carboxylic acid (acetic, lactic, tartaric, citric and malic), the weight loss was in the range 5.7–9.7% (Table 2). An explanation that can be given for this behaviour derives from the speciation of silicon atoms and other contaminants (Na, Mg, Ca, P, S, etc.) within the fibres of the carbon matrix of the rice husks. Regarding silicon in the cell walls of rice plant fibres, He et al. [18] found evidence of the presence of Si-hemicellulose complexes in which silicon crosslinks with the cis-diols polysaccharides of the cell walls, stabilizing them. The ligand nature of the polysaccharides on the silicon atoms (and presumably on the other contaminants) explains the extensive weight loss after treatment with strong acids since, at a low pH, the hydrolysis and denaturation of the heteroatom–ligand complexes are highly favoured compared to milder conditions.

**Figure 1:**
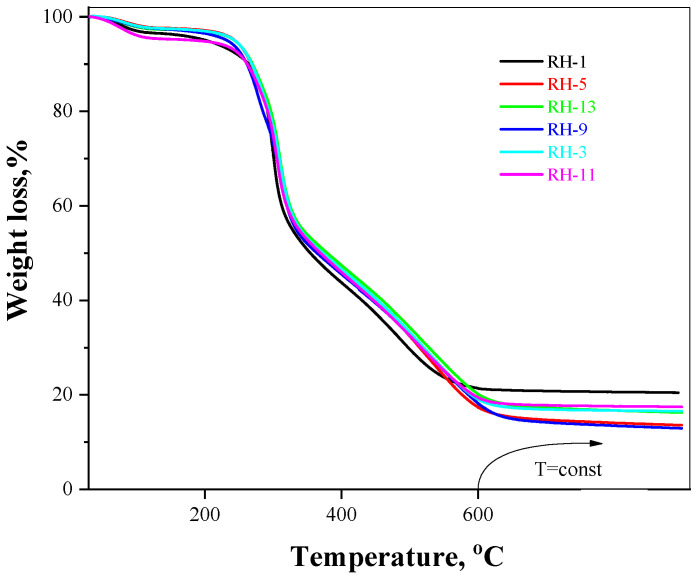
TG profiles of the decomposition of the organic components from rice husks, treated using carboxylic acids and HNO_3_.

The amount of organic components in the acid-treated samples was determined by thermogravimetric analysis under airflow (Figure 1, Table 2).

The method was also used to demonstrate the complete removal of the organic part in the material after calcination. According to Ref. [19] the nitric acid treatment was performed at 80 °C for 3 h. The weight loss during the acid treatment was 64.8 wt. %. The treatment with carboxylic acid was performed at 50 °C for 1 h, which resulted in weight loss between 5.7–9.7 wt. %. For the purpose of comparison, we performed an additional experiment of pretreatment with nitric acid at 50 °C for 1 h, showing lower weight loss (around 24.5 wt. %) than that at 80 °C for 3 h, but still far from the significantly low weight loss during pretreatment with carboxylic acid. Calcination is the next step of the procedure, which was performed at 500 °C and 600 °C, demonstrating the complete removal of the organic component, while during treatment at 400 °C, the remaining not decomposed organic component is 5–10%. Depending on the type of acid used for the pretreatment (mineral or carboxylic), differences in the yield of mesoporous silica are observed—7.2–7.6% yield after treatment with nitric acid and 12.8–16.5% after treatment with carboxylic acid (Table 2). Carboxylic acid treatment resulted in a higher yield of mesoporous silica.

The elemental composition of the impurities in the starting rice husks and in the resulting mesoporous silica (RH-11) were determined using an ICP-OES analysis (Table 3). The starting rice husks mainly contain calcium, potassium, magnesium, sulphur, phosphorus and manganese (>100 ppm). Results obtained for the final material indicate mesoporous silica with high purity (98.8 wt. %). The content of toxic elements is below 0.01% as oxides. A significant decrease in impurities, like Co, Pb, S, Na, Mg, Cu, B and Sr, was detected in the final material. After the acid and heat treatment, aluminium, iron and phosphorus were registered in the highest amounts as main constituents of the plant matrix (> 100 ppm) and remained in the obtained material after the performed treatments.

The high concentration of phosphorus with a fair amount of Al, Fe, Ca and Mg still present in the ash can be explained in much the same way as the speciation of silicon in its complexes with hemicellulose. Indeed, rice husks possess a high content of inositol hexaphosphate (IP6 or phytic acid), present as a mixed salt of Ca, K, Mg and capable of chelating Al, Fe and Zn ions [20] in different constituent parts of cereal crops. Although the high resistance to acid treatment of metal-IP6 complexes is high, making it undigestible by the gastric system and potentially toxic to non-ruminant mammals, the high calcination temperatures allow the decomposition of its carbonaceous component, generating stable metal phosphates as silica ash contaminants [21].

**Figure 2:**
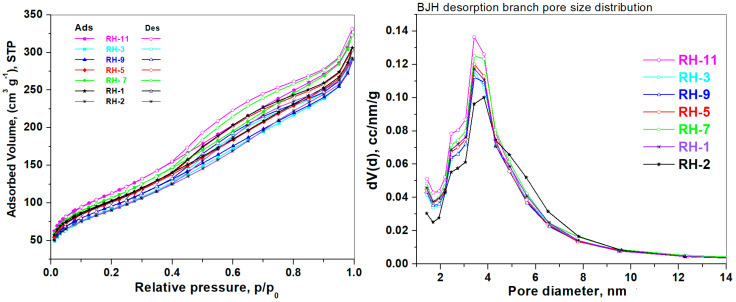
Nitrogen physisorption isotherms (**left**) and pore size distribution (**right**) of the mesoporous silicas obtained from rice husks treated using carboxylic acids and nitric acid.

**Figure 3:**
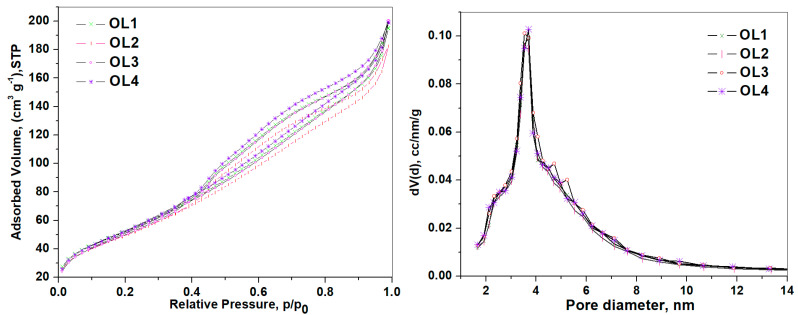
Nitrogen physisorption (**left**) and pore size distribution (**right**) of the obtained mesoporous silicas methodologically obtained through prior calcination (OL1 and 2) and posthumous treatment in nitric acid (OL3) or citric acid (OL4).

The obtained materials after calcination were characterized by nitrogen physisorption, scanning electron microscopy (SEM) and transmission electron microscopy (TEM). The textural characteristics of the obtained samples were determined using nitrogen physisorption (Figure 2 and Figure 3). The calculated parameters are presented in Table 2. The obtained isotherms are of type IV-a, characteristic of mesoporous materials with an H5 loop associated with certain pore structures containing both open and partially blocked mesopores [22]. The resulting mesoporous silica is distinguished by a high specific surface area of 300–420 m^2^/g and content of mesopores with a narrow size distribution and a maximum in the interval of 3.4–3.8 nm. The samples calcined at 500 °C show a higher specific surface area than those calcined at a higher temperature (600 °C), where further condensation of the silica matrix occurs. Treatment with carboxylic acids compared to treatment with nitric acid resulted in similar results for the specific surface area (336–420 m^2^/g for the material treated with carboxylic acids and calcined at 500 °C and 375 m^2^/g for the material treated with nitric acid and calcined at 500 °C).

It was found that the proposed procedure for treatment with carboxylic acids leads to the construction of a mesoporous structure with a narrower mesopore size distribution (2.7–4.4 nm) compared to the obtained material after treatment with nitric acid (2.4–5.6 nm) (Figure 2). Applying a different calcination temperature (500–600 °C) to the material obtained during treatment with carboxylic and mineral acids does not affect the pore size distribution of the obtained silica material, but only the specific surface area (Table 2). The only exception can be observed in the RH-2 sample treated with nitric acid and subsequently calcined at 600 °C (Figure 2, right panel), for which a broadening of the pore size distribution was recorded as a symptom of excessive breakdown of the original vegetable matrix, reflected in the silicon final product. The application of heating in a microwave reactor at different power (50 W and 100 W) during the acid treatment of rice husks resulted in a more than two-fold reduction of the treatment time (from 1 h to only 25 min). The lack of significant differences in the final samples obtained after treatment in a microwave reactor using different power (samples RH-13 and 14, Table 2) shows that the use of a power of 50 W is quite sufficient, thus maintaining the temperature of 50 °C during the acid treatment.

The advantages of the acid treatment directly on the rice husks instead of on the already calcined ashes are illustrated in Figure 3. Samples OL1–4, whose textural properties are illustrated in Table 2, were in fact prepared by calcining the rice husks at different temperatures and subsequently treating the latter with nitric or citric acid. Although this treatment best retains a particularly narrow pore distribution (Figure 3, right panel), a decrease of up to 50% of the exposed surface area was recorded in all samples of the OL series (Figure 2 left panel, Table 2).

**Scheme 1:**
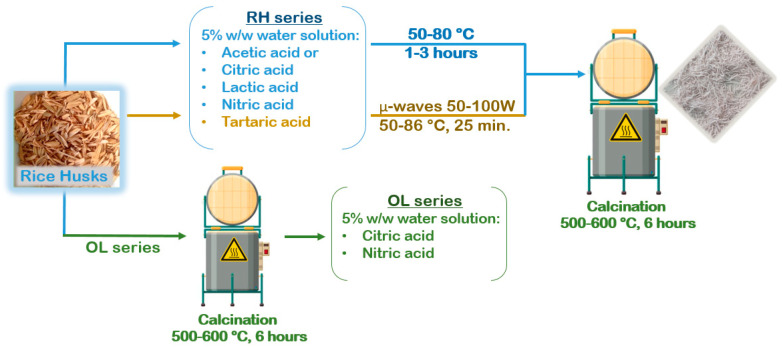
Diagram of the different synthetic procedures adopted in this study.

The combination of acid treatment and calcination of the rice husks resulted in the formation of a white powdery material. It is clearly seen that after acid treatment and removal of the organic component during calcination, the remaining silica component preserves the initial rice husks’ shape, thus indicating a homogenous silica distribution in the initial rice husks (Scheme 1). These observations confirm in a qualitative manner what has been said regarding the particular roles of cis-diols polysaccharides crosslinker and silicon in the cell walls of the plant. Hence, we can conclude that the organic cellular matrix of the rice husks serves as a natural template ensuring the formation of a mesoporous structure with narrow pore size distribution.

**Figure 4:**
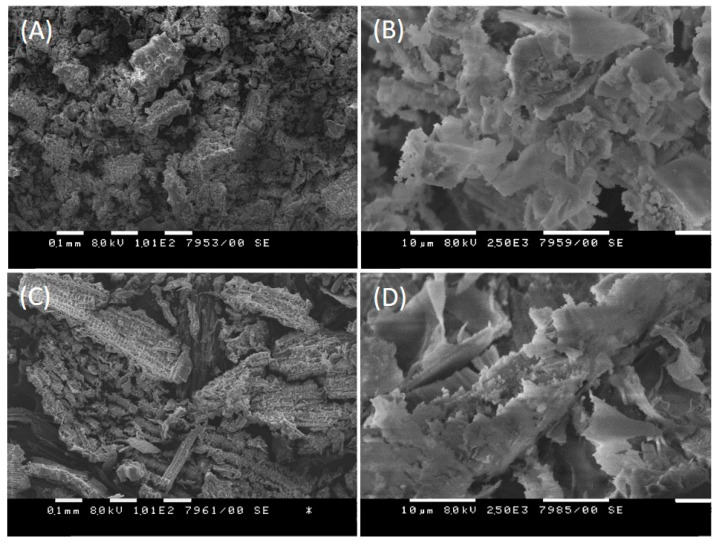
SEM micrographs of the sample treated with nitrogen acid RH-1 (**A**,**B**) and with citric acid RH-11 (**C**,**D**). The magnification of the micrographs at (**A**,**C**) is 100× and at (**B**,**D**)—2500×, respectively.

**Figure 5:**
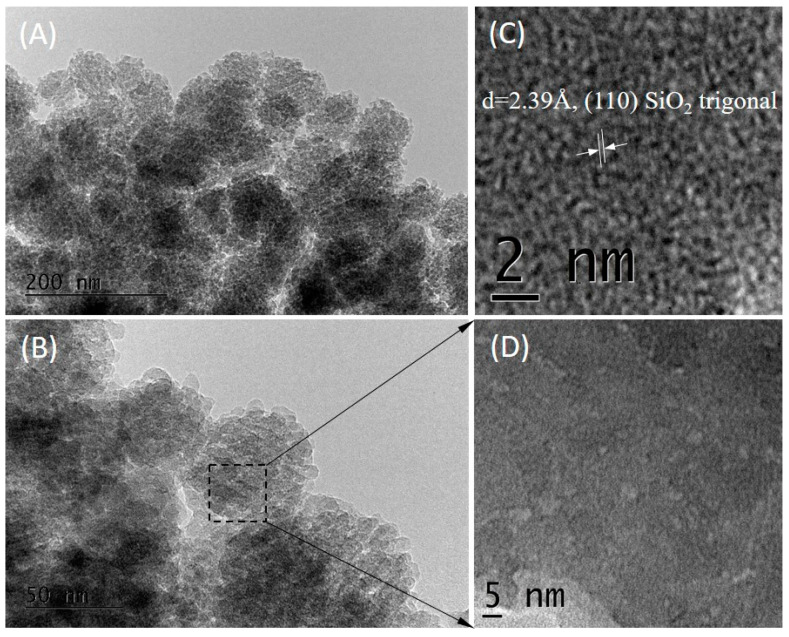
Bright Field ТЕМ micrographs of the mesoporous silicas obtained by treatment with citric acid at magnifications of 40 k (**A**) and 100 k (**B**). High-Resolution TEM micrograph of the same sample (**C**) with the visualization of the interplanar distance of (110) silica family plains. Square area in (**B**) magnified to 300,000× and presented in (**D**) separately for better visualization of the porous structure.

The morphology of the mesoporous silica investigated by SEM is presented at two magnifications in Figure 4 for the sample treated with nitric acid (A and B) and with citric acid (C and D). From the micrographs in Figure 4C,D, it is found that under the action of carboxylic acids (citric in this case) as well as nitric acid (Figure 4A,B), the integrity of the rice husks is destroyed as a result of the acid treatment and the subsequent calcination procedure. It is established also that parts of the disintegrated husk membrane co-exist together with a granular mass, mainly due to the oxidation of the silicon contained in the rice cells [23]. TEM was used to investigate the microstructure of this matter at the nanoscale level, the results of which are presented in Figure 5 for the sample treated with citric acid. The morphology, consisting of tiny grains forming sphere-like aggregates with sizes of 20–100 nm, distinguished by their porosity and typical mesoporous silicates, is visualized in Figure 5A,B at magnifications 40,000× and 100,000×. According to the synthesis procedure, the mesoporous silica is amorphous, but some nanodomains with a crystalline structure could be found (Figure 5C). The crystallinity permits applying the High-Resolution (HR) TEM mode of the microscope, registering the crystalline plains and evaluating the corresponding interplanar distance. The comparison with the data from the Crystallography Open Database (COD) reveals the presence of the phase SiO_2_ trigonal with cell parameters a = 4.77360 Å c = 5.30100 Å, according to Entry #96-900-0778.

For a better illustration of the pores, a zoomed-in square area in (B) is presented in Figure 5D, and it is seen that the pores’ sizes range from 2 to 5 nm, which is consistent with the estimation of the size from nitrogen physisorption of this material.

**Figure 6:**
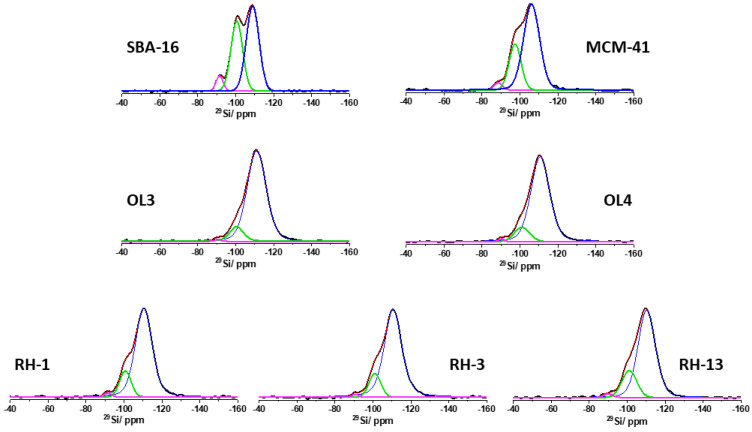
Direct excitation ^29^Si spectra of selected representative mesoporous silica materials obtained from rice husks (RH-1, RH-3, RH-13, OL3 and OL4) and standard mesoporous silica MCM-41 and SBA-16, shown for comparison. The experimental direct excitation ^29^Si spectra are given in black, while the simulated spectra are presented in red lines. The individual contributions of the different Si environments obtained after the deconvolution of the direct excitation ^29^Si spectra are given with coloured lines (Q^4^ blue, Q^3^ green, Q^2^ magenta).

Selected representative samples were characterized using solid-state NMR spectroscopy using direct excitation ^29^Si NMR spectra (Figure 6), providing quantitative information about the relative fractions of the different Si structural environments in the studied materials. The ^29^Si spectra of all samples show three main resonances that are characteristic of mesoporous silicas: Q^4^ (around −110 ppm, siloxane groups, [≡SiO)_4_Si]), Q^3^ (around −100 ppm, single silanol groups, [≡SiO)_3_SiOH]) and Q^2^ (around −91 ppm, geminal silanol groups, [≡SiO)_2_Si(OH)_2_]). The spectra were subjected to deconvolution in order to quantitatively analyze the relative fractions of the different Si environments (Table 4). All mesoporous materials were characterized by a very high fraction of Q^4^ units (84–88%) which constitute the main building blocks of the silica framework, while relatively low amounts of Q^3^ (11–15%) and Q^2^ (0.5–2%) type of silanol groups were detected.

These data imply that unlike the typical mesoporous silicas, such as MCM-41 and SBA-16, that are characterized by relatively high amounts of Q^3^ and Q^2^ silanol sites (last two rows in Table 4), the materials obtained from rice husks in our work have a highly ordered structure with a significant degree of crosslinking and with relatively small amount of defect silanol sites.

**Figure 7:**
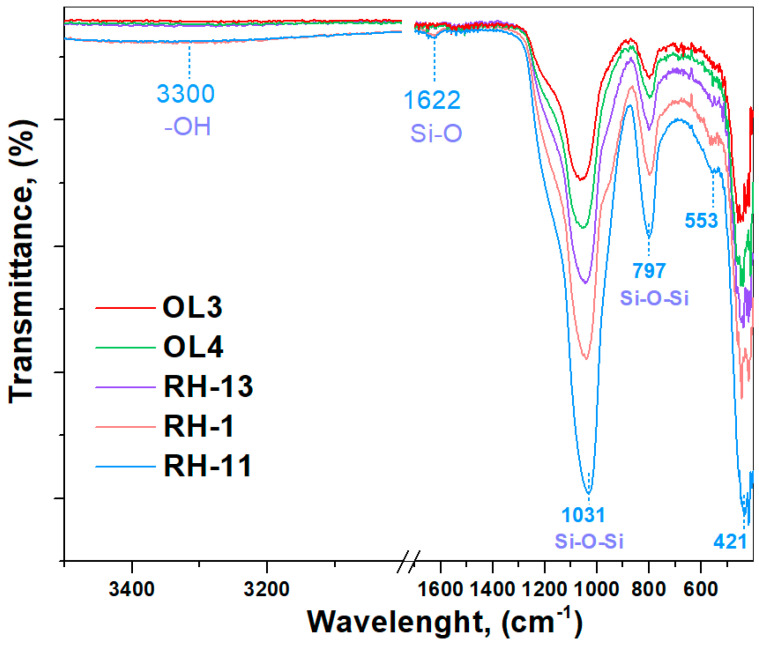
Overlay of ATR-FTIR spectra of selected samples of various rice husks after their acid treatment and annealing procedure.

The spectra of selected samples obtained by ATR-FTIR, shown in Figure 7, qualitatively represent what was previously demonstrated by ^29^Si solid-state NMR. In a completely complementary manner, the transmitted vibrational bands mainly consist of the asymmetric and symmetric, stretching and bending vibrations of the Si-O-Si bonds, respectively positioned at 1031, 797, 553 and 421 cm^−1^ [24]. The negligible presence of a broad band at wavenumbers higher than 3300 cm^−1^ and the related Si-O- vibrations in the region around 1600 cm^−1^ for all the samples under examination supports the analysis of the chemical shifts of the ^29^Si nuclei, demonstrating the preponderant composition of the SiO_2_ matrix mainly of siloxane groups rather than Q^3^ or Q^2^ silicon nuclei. Furthermore, no signals from residual organic molecules in the ATR-FTIR spectra were detected, an indication that the calcination temperatures used are fully effective in removing both the organic matrix and probably the carboxylic acid residues as suggested by the thermogravimetric measurements shown in Figure 1.

## 3. Conclusions

Mesoporous silica with worm-like porosity was obtained from rice husks by simple treatment with carboxylic acid followed by calcination at moderate temperatures (500–600 °C), that are high enough for the removal of the organic component. The variation of the nature of the applied carboxylic acids for rice husk treatment resulted in the formation of mesoporous silicas with similar textural characteristics, such as a specific surface area of 300–420 m^2^/g, a pore volume of 0.41–0.51 cm^3^/g and pores with a narrow size distribution and a maximum in the range of 3.4–3.5 nm. The formed mesopores have a volume of 0.37–0.46 cm^3^/g, corresponding to ~90% of the total pore volume and macropores, which are due to interparticle porosity, with a volume of 0.04–0.05 cm^3^/g, corresponding to ~10% of the total pore volume. Among the used carboxylic acids, the treatment with citric acid leads to the formation of mesoporous silicas with the highest surface area and pore volume. Additionally, the time for treatment could be shortened from 1 h to 25 min by applying the microwave treatment at 50 W. The formation of the mesoporous structure in the final materials was also revealed by TEM images. The high purity of the obtained mesoporous silica was detected using an ICP analysis (98.8 wt. %). The complete removal of the organic part of the rice husks in the final materials was shown using a thermogravimetric analysis and ATR-FTIR spectroscopy. The applied synthesis procedure resulted in the preparation of mesoporous silica materials which contain predominantly siloxane groups (≡SiO)_4_Si, 84–88%) as the main building blocks, which was proven using a solid-state NMR. The morphology of the mesoporous silicas originating from the rice husks was investigated by SEM which showed the disintegration of the husk membrane after the acid treatment and the presence of a granular mass, mainly due to the oxidation of the silicon contained in the rice cells. The developed procedure that involves the pretreatment of the rice husks with carboxylic acid is more environmentally friendly and economically efficient as it results in a higher yield of mesoporous silica than the procedure that comprises the use of nitric acid. Furthermore, the pretreatment of the rice husks with acid before the calcination step leads to mesoporous silica materials with a significantly higher specific surface area. The obtained mesoporous silicas are appropriate for the development of adsorbents, catalysts, drug carriers, etc.

## 4. Experimental Part

### 4.1. Synthesis of Mesoporous Silica from Rice Husks

The rice husk was washed thoroughly with deionized water and then subjected to acid treatment with 5% carboxylic acids or HNO_3_ at 50 °C or 80 °C for 3 h. Then, the rice flakes were calcined at 500 °C for 6 h, with a 5 °C/min heating rate [25]. The samples were denoted as RH-X, X = 1–14 depending on the applied acids for treatment and calcination temperature (Table 1). The acid treatment of rice husks (RH6 and RH-8) was performed by applying 50 W or 100 W in a microwave (Table 1). Additionally, other samples were prepared without acid treatment, but calcined at 500 °C and 600 °C, and were denoted as OL-1 and OL-2, respectively. OL-3 and OL-4 samples were prepared from OL-2 by acid treatment with nitric acid and citric acid, respectively, after calcination. The initial rice husks were treated with nitric acid at 80 °C for 3 h and 50 °C for 1 h [19] for comparison with the material obtained after carboxylic treatment.

### 4.2. Textural and Other Physico-Chemical Characterization Methods

The specific surface area and total pore volume were determined from N_2_ physisorption isotherms collected at −196 °C using AUTOSORB iQ-C-MP-AG-AG (Quantachrome Instruments (Anton Paar brand), Ashland, VA, USA). Samples were pretreated at 150 °C before nitrogen adsorption under a vacuum for 15 h.

The thermogravimetric analysis (TGA) investigations were performed using a STA449F5 Jupiter-type instrument from NETZSCH Gerätebau GmbH (Selb, Germany). In a typical measurement, 20 mg of the sample was placed in a micro-balance alumina crucible and heated in a flow of air (50 cm^3^/min) up to 500 or 600 °C with a rate of 5 °C/min and a final hold-up of 1 h.

The NMR spectra were recorded on a Bruker Avance III HD 600 NMR spectrometer (Bruker, Karlsruhe, Germany) operating at 599.98 MHz ^1^H frequency (119.21 MHz for ^29^Si), using a 4 mm solid-state i-CP/MAS dual ^1^H/^31^P-^15^N probe head. The samples were packed in 4 mm rotors (Zr_2_O) and spun at a magic angle spinning (MAS) rate of 10 kHz. The quantitative direct excitation ^29^Si NMR spectra were acquired with a single-pulse sequence, 90° pulse length of 4.5 s, time domain data points of 3 K, spectrum width of 29 kHz and the minimum 1000 transients were accumulated with a relaxation delay of 60 s between each scan. The spectra were zero-filled to 16 K data points and processed with an exponential window function (line broadening factor 10) before the Fourier transformation.

The morphological study of the obtained mesoporous silica on two different scales—micrometric and nanometric—was carried out using scanning (SEM) and transmission electron microscopy (TEM), respectively. For this purpose, a Philips 515 (Eindhoven, The Nederlands) digitized scanning electron microscope operating at an 8 kV accelerating voltage and a JEOL JEM 2100 (JEOL Ltd., Tokyo, Japan) transmission electron microscope at 200 kV were used.

In the samples’ preparation procedure for SEM, the powders were fixed on the special microscope holders by means of conductive carbon tape and then covered in a sputter coater with a layer of Au-Pd deposited on their surface. For transmission microscopy, a suspension in ethanol was initially prepared from the powder sample. A drop of it was then fixed on a standard copper TEM grid coated with amorphous carbon and dried at room temperature.

ATR–FTIR spectra were recorded by means of an IRAffinity-1 “Shimadzu” Fourier-Transform Infrared (FTIR) spectrophotometer (Shimadzu, Japan) with a MIRacle Attenuated Total Reflectance Attachment. The instrument was equipped with a temperature-controlled, high-sensitivity DLATGS detector and ATR attachment with a KRS-5 prism. In general, 50 scans and a 4 cm^−1^ resolution were applied. The spectral data were processed with IR solution software.

Elemental determination in the initial rice husks and the obtained silica was performed by ICP-OES. About 0.5 g of samples were weighed and transferred into Teflon vessels of a microwave digestion system. A mixture of 6 mL 67% HNO_3_, 2 mL 30% H_2_O_2_ and 3 mL HF was added, and the samples were left overnight. Then, the samples were digested using a microwave digestion procedure, programmed as follows: 10 min to reach 180 °C and 15 min maintained at this temperature. The vessels were cooled, placed on a sand bath and carefully evaporated to near dryness. Finally, 10 mL of 1 M nitric acid was added and the solutions were transferred to a 25 mL volumetric flask and diluted to the 50 mL mark with deionized water. The element concentrations were measured by ICP-OES (Varian ICP-OES Vista Pro, Agilent (HP), Markham, ON, Canada) under optimal instrumental parameters. Three parallel subsamples were analyzed from each sample with the arithmetic mean and standard deviation calculated. A blank sample was passed through the whole analytical procedure.

## Figures and Tables

**Table 1:** Preparation procedure and characteristics of waste-derived mesoporous silica.

Conditions of Pretreatment of Rice Husks	Conditions of Heat-Treatment	Properties (SA: Surface Area, PV: Pore Volume)	Reference
No pretreatment	600 °C for 5 h	SA = 36.9 m^2^/g PV = 0.094 cm^3^/g	[7]
Averrhoa juice bilimbi (1 h at 80 °C)	500 °C for 6 h	SA = 204 m^2^/g	[8]
HCl solution (8 wt. %), refluxing for 4 h	610 °C for 2–3 h	SA = 204–352 m^2^/g PV = 0.35–0.52 cm^3^/g	[9]
1 M HCl, 80 °C, 2 h	500 °C for 4 h	SA = 46–264 m^2^/g	[10]
Gluconic and citric acids (at 80 °C for 2 h, at acid concentrations 0; 0.05; 0.15; 0.25; 0.35 mol/L)	800 °C for 2 h	SA = 114.06 m^2^/g PV = 0.23 cm^3^/g	[11]
Ionic liquid (1-butyl-3-methylimidazolium chloride) at 100–150 °C for 12–36 h	700 °C for 2 h	SA = 241.1 m^2^/g	[12]
5.0 wt. % HCl for 1 h.	700 °C for 2 h	SA = 283.3 m^2^/g	[12]
Acidic solution of HCl (0.01–1.00 M) for 1.5 h	500 °C, 700 °C, 1000 °C, rate 2 °C/min, 5 °C/min, 10 °C/min	SA: 98.41–153.10 m^2^/g PV: 0.00498–0.00650 cm^3^/g	[13]
Acidic solution of HNO_3_, 0.1 M for 1.5 h	700 °C, rate 5 °C/min	SA = 129.80 m^2^/g PV = 0.00610 cm^3^/g	[13]
Acidic solution of CH_3_COOH 0.1 M for 1.5 h	700 °C, rate 5 °C/min	SA = 13.40 m^2^/g PV = 0.00567 cm^3^/g	[13]

**Table 2:** Procedure, yield and textural characteristics of the obtained mesoporous silicas.

Samples	Preparation Conditions	T_calc._, °C	Weight Loss after Acid Treatment, wt. %	Weight Loss after Calcination, wt. %	Yield, %	S_BET_, (m^2^/g)	Total Pore Volume, (cc/g)	Pore Sizes, (nm)
RH-1	Nitric acid, 80 °C, 3 h	500	64.8	78.5	7.6	375	0.48	3.4
RH-2	Nitric acid, 80 °C, 3 h	600	64.8	79.5	7.2	329	0.45	3.8
RH-3	Acetic acid, 50 °C, 1 h	500	9.7	83.1	15.3	336	0.47	3.4
RH-4	Acetic acid, 50 °C, 1 h	600	9.7	84.7	13.8	302	0.44	3.4
RH-5	Lactic acid, 50 °C, 1 h	500	9.6	83.8	14.6	367	0.47	3.4
RH-6	Lactic acid, 50 °C, 1 h	600	9.6	84.8	12.8	325	0.43	3.4
RH-7	Tartaric acid, 50 °C, 1 h	500	9.7	83.2	15.2	390	0.50	3.4
RH-8	Tartaric acid, 50 °C, 1 h	600	9.7	84.8	13.7	348	0.45	3.4
RH-9	Malic acid, 50 °C, 1 h	500	9.0	85.5	13.2	349	0.45	3.4
RH-10	Malic acid, 50 °C, 1 h	600	9.0	86.1	13.2	308	0.41	3.4
RH-11	Citric acid, 50 °C, 1 h	500	5.7	82.5	16.5	420	0.51	3.4
RH-12	Citric acid, 50 °C, 1 h	600	5.7	82.7	16.3	355	0.45	3.4
RH-13	Tartaric acid, MW at 50 W at 50 °C for 25 min	500	6.7	83.3	15.6	368	0.46	3.5
RH-14	Tartaric acid, MW at 100 W at 86 °C for 25 min	500	6.5	83.1	15.8	363	0.46	3.5
OL1	-	500	-	81.3	18.7	193	0.30	3.7
OL2	-	600	-	81.7	18.3	182	0.28	3.5
OL3	Nitric acid	-	-	-	-	199	0.31	3.7
OL4	Citric acid	-	-	-	-	205	0.31	3.5

**Table 3:** Content of impurities in the initial rice husks and oxides content in the obtained mesoporous silicas.

Elements	Content in Rice Husks, ppm	Oxide	Oxide Content in R-11, %
Co	1.2	CoO	<0.01
Аs	0.15	As_2_O_5_	<0.01
Pb	0.34	PbO	<0.01
S	290	SO_3_	0.02
Al	77	Al_2_O_3_	0.26
Na	91	Na_2_O	0.02
Mg	346	MgO	0.015
Cu	5	CuO	<0.01
B	4	BO_3_	0.01
Sr	3	SrO	0.01
Li	4	Li_2_O	<0.01
Ba	6	BaO	0.01
Ca	1104	CaO	0.02
Cd	0.12	CdO	0.1
Cr	3.4	Cr_2_O_3_	<0.01
Fe	62	Fe_2_O_3_	0.13
K	2661	K_2_O	0.04
Mn	154	MnO	0.02
Ni	0.45	NiO	0.06
P	239	P_2_O_5_	0.41
Zn	17	ZnO	<0.01
		SiO_2_	98.8

**Table 4:** Relative fractions of the different silica structural units are calculated by the deconvolution of the direct excitation ^29^Si NMR spectra.

Sample	Q^2^		Q^3^		Q^4^	
	ppm	%	ppm	%	ppm	%
RH-1	−91.5	1.0	−100.7	15	−110.4	84.0
RH-3	−90.7	1.0	−101.0	14.0	−110.6	86.0
RH-13	−90.8	2.0	−100.2	13.0	−109.9	85.0
OL3	−90.7	0.5	−100.4	11.0	−111.0	88.5
OL4	−90.4	1.0	−101.1	11.0	−110.7	88.0
MCM-41	−90.8	3	−100.3	27	−109.8	70
SBA-16	−91.4	5	−100.5	41	−108.9	54

## Data Availability

All data are available from the authors.

## References

[1] Kresge C.T., Leonowicz M.E., Roth W.J., Vartuli J.C., Beck J.S. (1992). Ordered mesoporous molecular sieves synthesized by a liquid-crystal template mechanism. Nature.

[2] Schuth F. (2001). Non-siliceous Mesostructured and Mesoporous Materials. Chem. Mater..

[3] Slowing I.I., Vivero-Escoto J.L., Trewyn B.G., Lin V.S.-Y. (2010). Mesoporous silica nanoparticles: Structural design and applications. J. Mater. Chem..

[4] Baerns M. (2003). Basic Principles in Applied Catalysis.

[5] Corma A. (1997). From Microporous to Mesoporous Molecular Sieve Materials and Their Use in Catalysis. Chem. Rev..

[6] Gebretatios A.G., Pillantakath A.R.K.K., Witoon T., Lin J.-W., Banat F., Cheng C.K. (2023). Rice husk waste into various template-engineered mesoporous silica materials for different applications: A comprehensive review on recent developments. Chemosphere.

[7] Araichimani P., Prabu K.M., Kumar G.S., Karunakaran G., Surendhiran S., Shkir M., Al Faify S. (2022). Rice Husk-Derived Mesoporous Silica Nanostructure for Supercapacitors Application: A Possible Approach for Recycling Bio-Waste into a Value-Added Product. Silicon.

[8] Midhun Dominic C.D., Neenu K.V., Sabura Begum P.M., Joseph R., dos Santos Rosa D., Duan Y., Balan A., Ajithkumar T.G., Soumya M., Shelke A. (2023). Nanosilica from Averrhoa bilimbi juice pre-treated rice husk: Preparation and characterization. J. Clean. Prod..

[9] Gu S., Zhou J., Yu C., Luo Z., Wang Q., Shi Z. (2015). A novel two-staged thermal synthesis method of generating nanosilica from rice husk via pre-pyrolysis combined with calcination. Ind. Crops Prod..

[10] Morales-Paredes C.A., Rodríguez-Linzán I., Saquete M.D., Luque R., Osman S.M., Boluda-Botella N., Joan Manuel R.-D. (2023). Silica-derived materials from agro-industrial waste biomass: Characterization and comparative studies. Environ. Res..

[11] Setiawan W.K., Chiang K.-Y. (2021). Eco-friendly rice husk pre-treatment for preparing biogenic silica: Gluconic acid and citric acid comparative study. Chemosphere.

[12] Chen H., Wang W., Martin J.C., Oliphant A.J., Doerr P.A., Xu J.F., DeBorn K.M., Chen C., Sun L. (2013). Extraction of Lignocellulose and Synthesis of Porous Silica Nanoparticles from Rice Husks: A Comprehensive Utilization of Rice Husk Biomass. ACS Sustain. Chem. Eng..

[13] Rafiee E., Shahebrahimi S., Feyzi M., Shaterzadeh M. (2012). Optimization of synthesis and characterization of nanosilica produced from rice husk (a common waste material). Int. Nano Lett..

[14] Nzereogu P.U., Omah A.D., Ezema F.I., Iwuoha E.I., Nwanya A.C. (2023). Silica extraction from rice husk: Comprehensive review and applications. Hybrid Adv..

[15] Steven S., Restiawaty E., Bindar Y. (2021). Routes for energy and bio-silica production from rice husk: A comprehensive review and emerging prospect. Renew. Sustain. Energy Rev..

[16] Adam F., Appaturi J.N., Iqbal A. (2012). The utilization of rice husk silica as a catalyst: Review and recent progress. Catal. Today.

[17] Peralta Y.M., Molina R., Moreno S. (2024). Chemical and structural properties of silica obtained from rice husk and its potential as a catalytic support. J. Environ. Chem. Eng..

[18] He C., Wang L., Liu J., Liu X., Li X., Ma J., Lin Y., Xu F. (2013). Evidence for ‘silicon’ within the cell walls of suspension-cultured rice cells. New Phytol..

[19] Andas J., Ekhbal S.H., Ali T.H. (2021). MCM-41 modified heterogeneous catalysts from rice husk for selective oxidation of styrene into benzaldehyde. Environ. Technol. Innov..

[20] Wu P., Tian J.-C., Walker C.E., Wang F.-C. (2009). Determination of phytic acid in cereals—A brief review. Int. J. Food Sci. Technol..

[21] Daneluti M., Jivaldo A., Jivaldo M. (2013). Study of thermal behavior of phytic acid. Braz. J. Pharm. Sci..

[22] Thommes M., Kaneko K., Neimark A.V., Olivier J.P., Rodriguez-Reinoso F., Rouquerol J., Sing K.S.W. (2015). Physisorption of gases, with special reference to the evaluation of surface area and pore size distribution (IUPAC Technical Report). Pure Appl. Chem..

[23] Shrestha D., Nayaju T., Kandel M.R., Pradhananga R.R., Park C.H., Kim C.S. (2023). Rice husk-derived mesoporous biogenic silica nanoparticles for gravity chromatography. Heliyon.

[24] Ellerbrock R., Stein M., Schaller J. (2022). Comparing amorphous silica, short-range-ordered silicates and silicic acid species by FTIR. Sci. Rep..

[25] Mitova V., Tsacheva I., Rosmini C., Dimitrov M., Popova M., Koseva N. “Mesoporous silica from rice husks”, utility model 4564/06.11.2023 г.

